# Physiological Profile Assessment of Posture in Children and Adolescents with Autism Spectrum Disorder and Typically Developing Peers

**DOI:** 10.3390/brainsci10100681

**Published:** 2020-09-27

**Authors:** Cecilia Perin, Giulio Valagussa, Miryam Mazzucchelli, Valentina Gariboldi, Cesare Giuseppe Cerri, Roberto Meroni, Enzo Grossi, Cesare Maria Cornaggia, Jasmine Menant, Daniele Piscitelli

**Affiliations:** 1 School of Medicine and Surgery, University of Milano Bicocca, 20126 Milan, Italy; giulio.valagussa@gmail.com (G.V.); miryam.mazzucchelli@gmail.com (M.M.); valentina.gariboldi@gmail.com (V.G.); cesare.cerri@unimib.it (C.G.C.); cesaremaria.cornaggia@unimib.it (C.M.C.); daniele.piscitelli@mcgill.ca (D.P.); 2 Autism Research Unit, “Villa Santa Maria” Foundation, 22038 Como, Italy; Enzo.Grossi@bracco.com; 3 ASST Rhodense, Ospedale “G. Salvini”, 20024 Milan, Italy; 4 Department of Physiotherapy, LUNEX International University of Health, Exercise and Sports, Differdange, 4671 Luxembourg, Luxembourg; roberto.meroni@lunex-university.net; 5 Neuroscience Research Australia and School of Public Health and Community Medicine, University of New South Wales, Sydney, NSW 2052, Australia; j.menant@neura.edu.au; 6 School of Physical and Occupational Therapy, McGill University, Montreal, QC H3G 1Y5, Canada; 7 School of Physical Therapy and Athletic Training, Pacific University, Hillsboro, OR 97123, USA

**Keywords:** autism spectrum disorder, neurodevelopmental disorders, assessment, sensorimotor integration, postural balance

## Abstract

A sound postural system requires sensorimotor integration. Evidence suggests that individuals with Autism Spectrum Disorder (ASD) present sensorimotor integration impairments. The Physiological Profile Assessment (PPA) can be used to evaluate postural capacity assessing five physiological subsets (i.e., vision, reaction time, peripheral sensation, lower limb strength, balance); however, no studies applied the PPA in young individuals. Therefore, this study aimed to investigate the PPA in children and adolescents with ASD compared with age-matched typically developing (TD) individuals and examine the relationship between the PPA subset within the ASD and TD participants according to different age groups. Percentiles from the PPA were obtained from the TD children and adolescents (*n* = 135) for each test. Performances of the individuals with ASD (*n* = 18) were examined relative to the TD percentiles. ASD participants’ scores were above the 90th percentile (i.e., poor performance) in most sensory, motor and balance parameters. Performance in most of the PPA tests significantly improved with older age in the TD group but not in the ASD group. The study findings support the use of the PPA in TD children and adolescents while further research should investigate postural capacity in a larger ASD sample to enhance the understanding of sensorimotor systems contributing to compromised postural control.

## 1. Introduction

Autism spectrum disorder (ASD) is a neurodevelopmental disorder affecting ~1 in 59 with four males diagnosed for each female in North America [1] and 1 in 87 children aged 7–9 years in Italy [2]. According to the Diagnostic and Statistical Manual of Mental Disorders fifth edition (DSM-5) [3], individuals with ASD are diagnosed based on some core features that manifest during the early developmental years. Deficits in social communication and social interaction are associated with repetitive patterns of behavior, interests or activities that cause significant impairment in social, occupational or other areas of functioning. In addition to these characteristic features, several motor dysfunctions have been described in ASD children and teenagers including disruptions in motor milestone development [4,5], clumsiness, impaired motor coordination, disturbance in reach-to-grasp movements [6,7,8], deficits in gross and fine motor skills [9] and abnormal gait patterns [10,11] Postural control impairments have also been reported in ASD individuals [12,13,14,15,16,17].

Postural control is a fundamental skill in daily human life for the ability to plan the coordination of movement and in maintaining dynamic and static balance for social interactions [18]. Postural control relies on the sensorimotor integration of various sources such as vestibular, visual and somatosensory pathways and actual states [19]. Interestingly, children and adolescents with ASD appear to have sensory processing that differentiate them from typically developing (TD) peers [20,21,22]. Individuals with ASD exhibited deficits in functional balance and motor performance [14].

To date, studies of postural control in children with ASD have used standardized clinical tests to assess gross motor proficiency in children such as the Movement Assessment Battery for Children-2 (MABC-2) [23] and the Test of Gross Motor Development [24], the Zurich Neuromotor Assessment [25] and the Physical Neurological Examination of Subtle Signs (PANESS) [26] or force platform instrumental approaches [13,27,28,29]. However, these tests have not integrated the multiple features of sensorimotor parameters involved in postural control for the maintenance of balance.

Lord et al. [30] developed the Physiological Profile Assessment (PPA) for a comprehensive assessment of sensorimotor functions related to postural capacity. The PPA assesses five physiological systems, i.e., vestibular function, peripheral sensation, muscle force, vision and reaction time, which are involved in the maintenance of stable and dynamic balance.

The PPA has been used on healthy individuals [31], subjects with neurological disorders to assess postural impairments [32,33,34] and the elderly with musculoskeletal disorders [35]. This instrument, based on interval scales [36], has been demonstrated to have psychometrically sound proprieties with a lower administrative burden compared with biomechanical assessments using motion capture and force platforms [30,33].

Although the PPA was successfully administrated in the adult population, no studies investigated its applicability in younger individuals. Therefore, the first aim of this cross-sectional study was to examine the sensory and motor functions involved in postural control in TD children and adolescents and in age-matched individuals with ASD using the PPA. The second aim was to investigate the relationship between each PPA subset within ASD and TD participants for each age group. The preliminary results were presented in abstract form [37].

## 2. Materials and Methods

### 2.1. Participants

TD individuals were recruited from three public schools in the suburbs of a metropolitan city in northern Italy. Children and adolescents with ASD were recruited from a Neuropsychiatric Institute where ASD patients were followed. Inclusion criteria were as follows: age between 6 and 18 years and an ASD diagnosis according to the DSM-5 criteria [3] confirmed through an Autism Diagnostic Observation Schedule (ADOS-2) [38]. Exclusion criteria were neurological or orthopedic co-morbidities that might influence the test performances, lack of compliance with the PPA, interruption of the assessments and/or showing signs of irritation. Control participants (TD) were typically developing children and adolescents aged between 6 and 18 years. Exclusion criteria were neurological, cognitive or orthopedic impairments that might influence performance in the sensorimotor and balance tests (e.g., the ability to sit, stand and ambulate independently).

The study was conducted following the declaration of Helsinki and was approved by the local Ethics Committee (Protocol Number: 3532017). All parents provided written, informed consent to allow their child’s participation in the study.

### 2.2. Physiological Profile Assessment

All ASD and TD children and adolescents were assessed using the PPA [30]. The PPA is a multi-item instrument that evaluates physiological domains contributing to postural control. The PPA includes tests of vision, peripheral sensation, lower limb muscle strength, simple reaction time and balance, which are detailed below. The assessment for ASD children was performed by two residents in physical medicine and a rehabilitation clinician. TD children were assessed by six trained residents in physical medicine and a rehabilitation clinician. All assessors were trained to administer the PPA. The clinicians were not blind when performing the PPA with ASD and TD participants. The researchers involved in the data analysis were blind.

#### 2.2.1. Vision

Visual acuity was measured using a letter chart with high- and low-contrast (10%) letters. Acuity was assessed binocularly with participants wearing their distance glasses (if applicable) at a test distance of 3 m and measured in terms of the logarithm of the minimum angle resolvable in minutes of arc (logMAR). Edge contrast sensitivity was assessed using the Melbourne Edge Test, which presents 20 circular patches containing edges with reducing contrast. The lowest contrast patch correctly identified was recorded as the participant’s contrast sensitivity in decibel units where one dB = 10 log10 contrast. Depth perception was measured using a Howard-Dohlman depth perception apparatus. This device presents two vertical rods; one is fixed and the other one can move forward and back along a track using two strings. Participants are required to pull on the strings to adjust the position of the movable rod to align it to the fixed one. Participants are seated three meters away from the apparatus. The error in aligning the rods is recorded in centimeters. The average of four trials was computed.

#### 2.2.2. Peripheral Sensation

Tactile sensitivity on the dominant ankle was measured with a Semmes-Weinstein-type pressure aesthesiometer. The filaments were applied to the center of the lateral malleolus of the ankle. Participants were instructed that the filament would be placed on their ankle when the examiner said “A” or “B.” If they felt the filament in contact with their skin, they had to report to the examiner whether they felt it on “A” or “B.” The finest filament correctly detected was identified. Pressure (in grams) exerted by this filament was converted to log10 0.1 mg, yielding a scale of approximately equal intensity intervals between filaments. Proprioception was measured using a lower limb matching test that recorded the difference (in degrees) in matching the great toes on either side of a vertical transparent acrylic sheet inscribed with a protractor and placed between the legs. Participants were required to perform the test with their eyes closed. The mean error of five trials was computed.

#### 2.2.3. Reaction Time

Simple reaction time was assessed in milliseconds using a hand-held electronic timer and a light as the stimulus and depression of a switch by the finger and the foot as the responses. A modified computer mouse was used as the response box for the finger press task and a pedal switch was used for the foot press task. Five practice trials were undertaken followed by 10 experimental trials. The mean of these 10 trials was computed.

#### 2.2.4. Lower Limb Muscle Strength

Isometric knee extensor and flexor muscle strength were assessed on the dominant leg using a spring gauge. The force of the knee extensor and flexor muscles was measured with the participant sitting in a tall chair with a strap around the leg 10 cm above the ankle joint and the hip and knee joint angles positioned at approximately 90 degrees. In three trials per muscle group, the subject attempted to push/pull against the strap assembly with maximal force for 2 to 3 s; the greatest force for each muscle group was recorded in kilograms. Ankle isometric dorsiflexion strength was assessed using a footplate attached to a spring gauge. Participants were seated in a standard chair and their dominant foot was secured to the footplate with the angle of the knee at approximately 110 degrees. The greatest maximal dorsiflexion force (in kilograms) measured out of three trials was recorded.

#### 2.2.5. Balance

Participants performed the balance tests barefoot using a swaymeter that measured displacements of the body at the level of the waist. The device consisted of a 40 cm-long rod with a vertically mounted pen at its end. The rod was attached to the subject by a firm belt and extended posteriorly for the tests of postural sway and anteriorly for the tests of maximal balance range and coordinated stability. Postural sway was assessed with eyes open and closed; first on a firm surface then on a medium density foam rubber mat (15 cm thick). In each of the four conditions, the pen on the extremity of the swaymeter recorded the subject’s sway on a sheet of millimeter graph paper fastened to the top of an adjustable-height table as the subject attempted to stand as still as possible for 30 s. For each trial, anteroposterior and mediolateral sway were recorded in mm and the sway area (anteroposterior x mediolateral distances) was calculated in mm^2^. Limits of stability and control of the center of mass (COM) movements were assessed using the maximal balance range and coordinated stability tests. The maximum balance range test measured the limits of stability in the anteroposterior plane as the participants were instructed to lean as far forward and as far back as possible without moving the feet or bending at the hips. The test was repeated three times with the highest anteroposterior range taken as the test result. The score was obtained by multiplying the distance in mm by a factor that considered the average height per age [39] and the age of the subject. The coordinated stability test required each participant to adjust their balance by leaning or rotating the body without moving the feet so that the pen followed and remained within the borders of a 1.5 cm-wide convoluted track. A total error score was calculated by summing the number of occasions that the pen failed to stay within the path; five points were accrued for a cut corner and one for a crossed side. Participants performed one practice trial before the experimental trial. A score corresponding to mean plus three standard deviations calculated per age was attributed to the subjects unable to perform some subsets of the PPA according to Lord, Menz and Tiedemann [30].

### 2.3. Statistical Analysis

The TD participants were divided into eight age groups (i.e., one group per year) that matched the individual ages of the ASD participants: 6 years, 8 years, 11 years, 12 years, 13 years, 14 years, 16 years and 18 years old. Data normality was confirmed through the visual inspection of quantile-quantile (Q-Q) plots and Kolmogorov–Smirnov tests. Given the skewness of the data distribution, we used percentiles for each PPA item and age group to obtain a reference database of TD children and adolescents. We calculated the 10th, 50th and 90th percentiles for each performance and each age range. For some parameters (edge contrast sensitivity, lower limb strength, maximum balance range test), the scale was inverted so that all items scoring over the 90th percentile indicated poorer performance and those scoring under the 10th percentile indicated better performance. For each test, we compared scores of single ASD subjects with the scores obtained by TD subjects in the age-matched group. Spearman correlations were used to assess correlations between the PPA performance scores and age in each group separately. Data were analyzed using SPSS version 25 for Windows (SPSS, Inc., Chicago, IL, USA) and the *p*-value was set at <0.05.

## 3. Results

One hundred and thirty-five (100/35 male/female) TD children and adolescents were recruited according the following age groups: 6 years (*n* = 11), 8 years (*n* = 16), 11 years (*n* = 21), 12 years (*n* = 12), 13 years (*n* = 14), 14 years (*n* = 24), 16 years (*n* = 18) and 18 years (*n* = 19).

Eighteen age-matched children and adolescents with ASD were enrolled (age: 12.4 ± 3.7 years; 16/2 male/female). Participants with ASD were age-matched according the following groups: 6 years (*n* = 2), 8 years (*n* = 3), 11 years (*n* = 2), 12 years (*n* = 2), 13 years (*n* = 1), 14 years (*n* = 4), 16 years (*n* = 3) and 18 years (*n* = 1) old. Their intellectual disability ranged from mild (*n* = 5), moderate (*n* = 11) to severe (*n* = 2). Eight participants had an ASD severity level of 1, nine participants of 2 and one participant of 3. Analysis of the Autism Diagnostic Observation Schedule Calibrated Severity Score (ADOS CSS) showed five participants had a score in the Autism Spectrum Disorder range and 13 participants in Autism range. Detailed characteristics are shown in Table 1.

The test took between 60 and 90 min; two participants (ID 16 and 17) completed the assessment in two sessions. No TD participants or individuals with ASD reported fatigue during the assessment.

**Figure 1:**
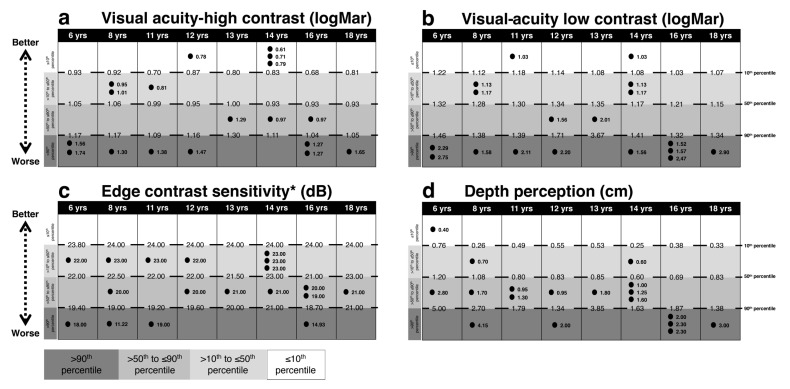
Comparison between each ASD subject’s performance in the Physiological Profile Assessment (PPA) vision tests and age-matched reference values (percentiles) for typically developing (TD) subjects. Light gray to dark gray corresponds with better to worse performance. In (**a**) visual acuity high-contrast, in (**b**) visual acuity low-contrast, in (**c**) edge contrast sensitivity, in (**d**) depth perception. The reference value of the 10th, 50th and 90th percentiles and each ASD subject values are reported. Intervals for each performance represent: ≤10th, 10th < x ≤ 50th, 50th < x ≤ 90th, >90th percentile from the top to the bottom, respectively. Abbreviations: yrs, years; LogMar, minutes of arc; dB, decibel units; cm, centimeters. * indicates the percentile scale was inverted, i.e., for all items a score over the 90th percentile is an indicator of a worse performance.

**Figure 2:**
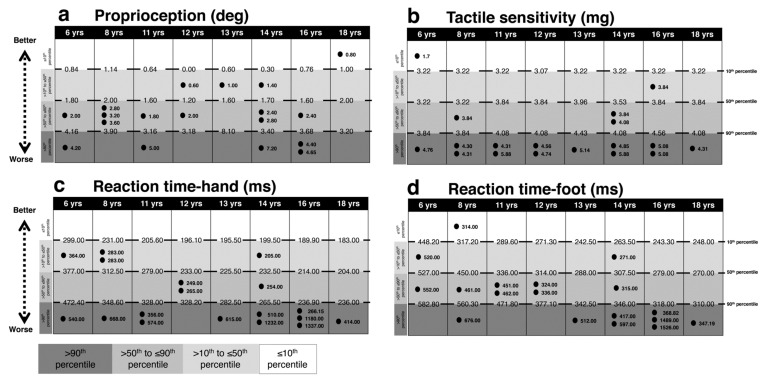
Comparison between each ASD subject’s performance in the PPA peripheral sensation and reaction time tests and age-matched reference values (percentiles) for TD subjects. Light gray to dark gray corresponds with better to worse performance. In (**a**) proprioception, in (**b**) tactile sensitivity, in (**c**) reaction time on hand, in (**d**) reaction time on foot. The reference value of the 10th, 50th and 90th percentiles and each ASD subject values are reported. Intervals for each performance represent: ≤10th, 10th < x ≤ 50th, 50th < x ≤ 90th, >90th percentile from the top to the bottom, respectively. Abbreviations: yrs, years; deg, degree; mg, milligrams; ms, milliseconds.

**Figure 3:**
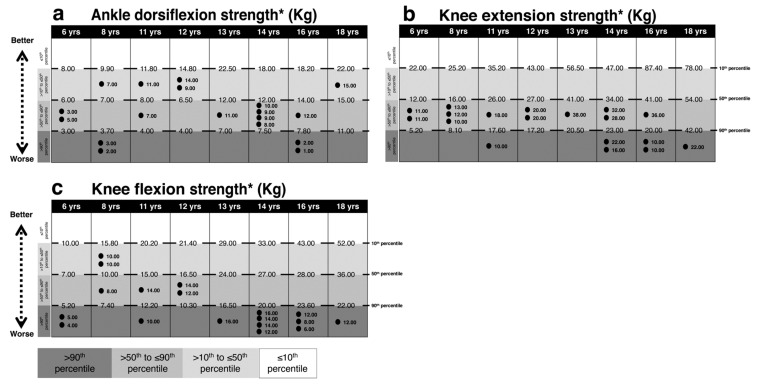
Comparison between each ASD subject’s performance in the PPA tests of lower limb strength and age-matched reference values (percentiles) for TD subjects. Light gray to dark gray corresponds with better to worse performance. In (**a**) ankle dorsiflexion force, in (**b**) knee extension force, in (**c**) knee flexion force. The reference value of the 10th, 50th and 90th percentiles and each ASD subject values are reported. Intervals for each performance represent: ≤ 10th, 10th < x ≤ 50th, 50th < x ≤ 90th, >90th percentile from the top to the bottom, respectively. * indicates the percentile scale was inverted, i.e., for all items a score over the 90th percentile is an indicator of a worse performance. Abbreviations: yrs, years; kg, kilograms.

**Figure 4:**
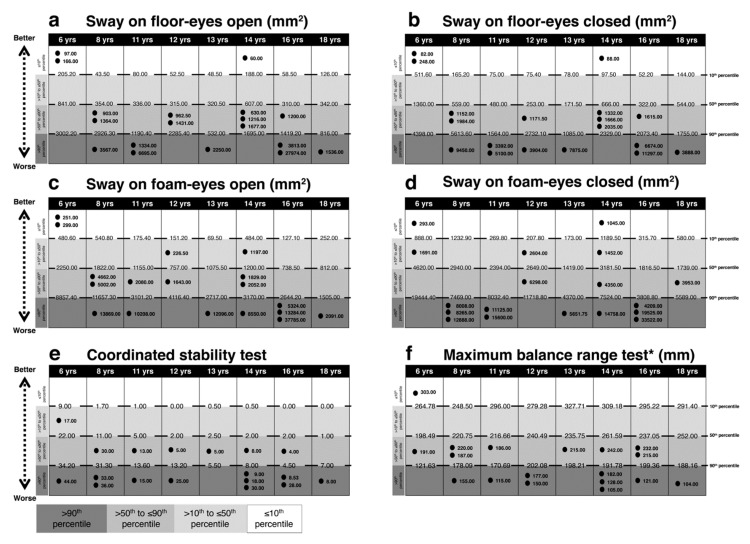
Comparison between each ASD subject’s performance in the PPA balance tests and age-matched reference value (percentiles) for TD subjects. Light gray to dark gray corresponds with better to worse performance. In (**a**) sway on floor eyes open, in (**b**) sway on floor eyes closed, in (**c**) sway on foam eyes open, in (**d**) sway on foam eyes closed, in (**e**) coordinated stability test, in (**f**) maximum balance range test. The reference value of the 10th, 50th and 90th percentiles and each ASD subject values are reported. Intervals for each performance represent: ≤10th, 10th < x ≤ 50th, 50th < x ≤ 90th, >90th percentile from the top to the bottom, respectively. * indicates the percentile scale was inverted, i.e., for all items a score over the 90th percentile is an indicator of a worse performance. Abbreviations: yrs, years; mm2, millimeters square; mm, millimeters.

Figure 1, Figure 2, Figure 3 and Figure 4 present performances of the ASD participants relative to the reference values (percentiles) of age-matched TD participants for vision, sensation, reaction time, force and balance tests, respectively. All percentiles for TD children and adolescents divided according to age groups can be found in Supplementary Table S1.

### 3.1. Vision

As shown in Figure 1, up to half of the ASD participants performed over the 90th percentile corresponding with a worse performance in the following tests: visual acuity high-contrast (*n* = 8), visual acuity low-contrast (*n* = 10), edge contrast sensitivity (*n* = 4) and depth perception (*n* = 6). Conversely, only a few ASD participants performed below the 10th percentile, indicating better performance in the following tests: visual acuity high-contrast (*n* = 4), visual acuity low-contrast (*n* = 2) and depth perception (*n* = 1).

Three-quarters of ASD subjects (*n* = 13) scored above the 90th percentile in the test of tactile sensitivity and one subject under the 10th percentile. In the test of proprioception, five subjects scored over the 90th percentile and one under the 10th percentile (See Figure 2).

### 3.2. Reaction Time

Eleven ASD individuals had prolonged hand reaction time over the 90th percentile relative to TD individuals and eight ASD individuals performed over the 90th percentile in the foot reaction time test. Of note though, one child with ASD had foot reaction times under the 10th percentile (Figure 2).

### 3.3. Lower Limb Muscle Strength

As displayed in Figure 3 regarding ankle dorsiflexor muscle strength, four subjects scored over the 90th percentile. For knee strength, six subjects scored over the 90th percentile for the knee extensor muscles and 12 subjects for the knee flexor muscles.

### 3.4. Balance

**Figure 5:**
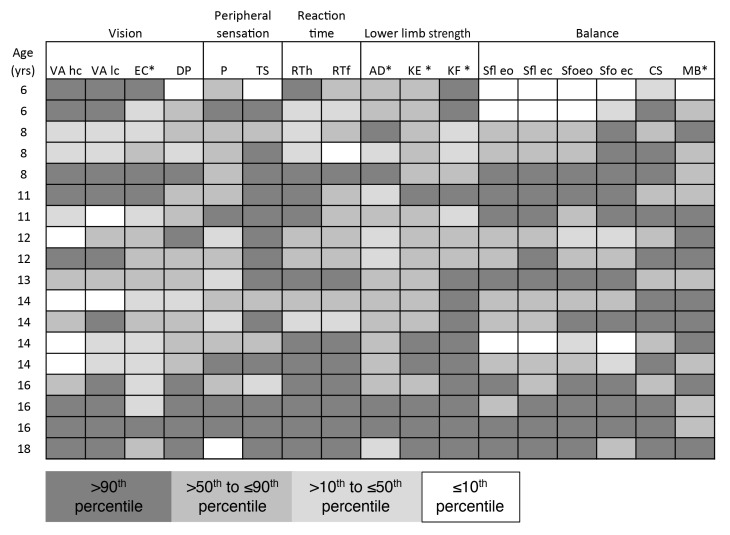
ASD subjects’ profile. Each ASD subject’s PPA performance box is colored with the correspondence of range percentile. Light gray to dark gray corresponds with better to worse performance. Abbreviations: yrs, years; VAhc, visual acuity high-contrast; VAlc, visual acuity low-contrast; EC, edge contrast sensitivity; DP, deep perception; P, proprioception; TS, tactile sensitivity; RTh, hand reaction time; RTf, foot reaction time; AD, ankle dorsiflexors force; KE, knee extensors force; KF, knee flexors force; Sfl eo, sway on floor eyes open; Sfl ec, sway on floor eyes closed; Sfo eo, sway on foam eyes open; Sfo ec, sway on foam eyes closed; CS, coordinated stability test; MB, maximum balance range test. * indicates the percentile scale was inverted so that all items scoring over the 90th percentile indicated poorer performance.

As shown in Figure 4, between seven and 10 subjects performed over the 90th percentile in the balance tests: sway on floor eyes open (*n* = 7), sway on floor eyes closed (*n* = 8), sway on foam eyes open (*n* = 8), sway on foam eyes closed (*n* = 10), coordinated stability test (*n* = 11) and maximum balance range test (*n* = 9). Conversely, three children performed under the 10th percentile for age in both tests of sway on the floor, two children in the test of sway on the foam with eyes open and two children in the test of sway on the foam with eyes closed and one child in the maximum balance range test. Figure 5 depicts the subjects’ profile according to the percentile for each of the PPA subtests. Notably, all ASD subjects showed poor performance at least in three subtests and all participants showed poor performance in one of the balance tests.

### 3.5. Relationships between Test Performance and Age

In TD participants, all items of the PPA significantly correlated with age except for edge contrast sensitivity, proprioception and sway on floor with eyes open and closed. In ASD participants, only performance in the test of coordinated stability was significantly correlated with age. Correlations are shown in Table 2.

## 4. Discussion

The results support the use of the five physiological measures of the PPA in TD children and adolescents. The PPA was also successfully administered to a subset of individuals with ASD during developmental ages. No fatigue and adverse events were reported from both groups.

We compared each PPA subtest between ASD and TD participants across age groups. Postural impairments might have a significant impact on the development of perceptual motor skills and social functioning in individuals with ASD [14]. According to Mergner’s multisensorial feedback model [40], an efficient postural control system requires the interaction of sensory, motor and integration systems. The central nervous system (CNS) integrates multiple inputs from visual, vestibular, proprioceptive and tactile somatosensory systems. The CNS includes both predictive and adaptive components whose regulation depends on a sensory reweight of the feedbacks enabling the human body to function correctly against gravity and environmental perturbation forces [19]. A marked deficit in any one of the feedback systems or a combination of mild impairments in multiple sensorimotor physiological domains may lead to postural disorders [18]. Sensorimotor organization mutates during human development [41] due to a variety of reasons including learning and aging processes that make individuals reach adult assets at around the age of 13–14 years old [42]. However, individuals with ASD appear to have patterns of sensory processing that differentiate them from TD peers [20,21,22].

As performances of the TD population in the sensory, motor and balance tests were entirely consistent, percentiles were used to represent the variables’ distribution. Our approach overcame the limitations of previous studies [14] by examining all components of balance systematically to obtain a complete profile of postural control in individuals with ASD.

Within our study, the presence of intellectual disability could not be ruled out to justify the different performances between TD subjects and participants with ASD. Therefore, the lower scores on the PPA items may be a consequence of an underlying intellectual disability or a combination with the presence of ASD. However, our sample was representative of a large subset of individuals with ASD. Notably, Newschaffer et al. [43] estimated that 70–75% of children with ASD have an intellectual disability.

### 4.1. Vision

According to a narrative review of literature by Bakroon and Lakshminarayanan [44], 29 to 44% of children and adolescents with ASD reported the presence of refractive errors. Here, half of our sample of children and adolescents with ASD performed poorly in the tests of visual acuity, particularly when the lighting conditions were poor (low-contrast). This result contrasts with the postulated superior visual acuity (eagle vision) of ASD subjects reported by some authors [45,46,47]. In fact, in our study only five ASD participants performed below the 10th percentile, which demonstrated a very high visual acuity performance. For static contrast sensitivity, our findings seemed to corroborate those presented in Simmons’ review [48] whereby there were no differences between ASD and TD performances. As also noted by Simmons et al. [48], depth perception or stereopsis was apparently preserved but has been scarcely studied; here, we found poor depth perception in more than a third of ASD participants. The reasons for the disagreement between the findings reported in the literature and ours may include different modalities of data collection (e.g., different tools to assess vision such as the Freiburg Acuity and Contrast Test) [45]; some authors also outlined the role of visual attention and its importance in visual integration [49].

### 4.2. Peripheral Sensation

Tactile sensitivity was inferior in our ASD population with three-quarters of participants scoring above the 90th percentile and one performing below the 10th percentile. Our results are in agreement with the literature [50,51] although a recent review questioned the high prevalence of tactile processing dysfunction in ASD [52]. It pointed out the difficulty to assess tactile sensitivity due to the subjectivity of clinical assessments, the heterogeneity of ASD cohorts and the diversity of tactile sensitivity measures; although it mostly concerns pain stimulation, another potential confounder to consider relates to emotional-affective conditioning in sensitivity tests [53]. Here, we used an aesthesiometer to be more accurate. One-quarter of ASD participants performed poorly in the test of proprioception. Similar to other sensory systems such as vision, the literature on the integrity of proprioception in ASD is conflicting. Some studies have reported altered proprioception [54] while others do not [55]; such discrepancies can be partly attributed to the variety of the test modality used. Notably, we tested proprioception in the leg, which has more relevance to standing postural control but is not as commonly investigated as in the upper limbs. In synthesis, the peripheral sensation was impaired in the ASD participants with most participants showing clear deficits in tactile sensitivity at the ankle and fewer underperforming in the proprioception assessment.

### 4.3. Reaction Time

We assessed simple reaction time with both the lower and upper limbs in individuals with ASD. More than half of our participants performed above the 90th percentile in the tests of simple reaction time at the finger and the foot, confirming previous reports of slow processing speed in ASD individuals [56]. Some studies [57,58,59] have also shown an increased response time variability in ASD but these works considered ASD subjects with Attention Deficit Hyperactivity Disorder. We might conclude that children and adolescents with ASD are slower compared with age-matched TD peers.

### 4.4. Lower Limb Muscle Strength

Over a quarter of the children and adolescents with ASD presented muscle weakness in at least one of the three lower limb muscle groups we investigated. Such deficits in muscle strength are likely to contribute to the altered gait patterns reported in ASD [60]. Interestingly, all of the adolescents with ASD aged 13 years and above presented evident knee flexor muscle weakness compared with age-matched TD peers. These older participants also included individuals with higher levels of intellectual disability and increased ASD severity. Interestingly, Kern et al. [61] reported that handgrip strength in children with ASD was related to the severity of the disorder.

### 4.5. Balance Tests

Our results for the sway tests with eyes closed are in line with most studies that showed increased postural sway of ASD subjects under these conditions [16,62,63]. Poor performance in the tests of coordinated stability and maximum balance range reflects the difficulty individuals with ASD have to control their center of mass at the limits of their stability [64]. Although the prevalence of balance problems in ASD is well acknowledged, explanations regarding the causes of the postural stability deficits diverge. For example, some authors outline the role of anxiety on postural instability [65] while others attribute the problem to poor cognition [66]. Nevertheless, most of the debate lies in whether postural deficits are related to sensorimotor or integrational problems. In fact, a body of literature suggests that rather than poor perceptive ability, failure in the integration of visual, somatosensory and vestibular information could be the primary contributors to poor balance [28]. While we agree that sensory integration needs to be considered, our findings demonstrate that poor performances in the single sensorimotor tests administered clearly impact on postural control in ASD.

### 4.6. Correlation with Age

The second aim of this work was to determine if the correlation between age and physiological parameters was similar for TD and ASD children and adolescents. Spearman correlation coefficients calculated for all variables showed a marked difference between the groups. In fact, TD participants’ performances improved with increasing age in most components of the PPA (except for edge contrast sensitivity, proprioception and sway on floor eyes open and closed) while for ASD participants, only the coordinated stability test performance was significantly correlated with age. Our findings support previous reports that tactile sensitivity [67], visual acuity [68] and reaction time [69] improve with increasing age in children and adolescents but disagree with those regarding contrast sensitivity [68] and proprioception [70,71]. In these latter studies, proprioception was assessed in the forearm while here proprioception was evaluated in the leg. To this end, our findings suggest that most balance-influencing parameters mature with age but not in ASD.

### 4.7. Limitations

We acknowledge that our work has some limitations. The first is that we did not collect the intelligence quotient for all participants in this study. We therefore cannot make firm conclusions regarding the potential contribution of cognition on sensorimotor integration and balance in the ASD sample. The second limitation involves the significant amount of interpersonal interactions between the participant and the examiner required to conduct the PPA, which might have affected the testing given communication issues are inherent to ASD. Third, the duration of the PPA could also deter individuals from participating. Finally, the reduced sample sizes of the age-matched individuals with ASD may affect the generalizability of the postural findings to the ASD population. Moreover, we recommend that future studies should investigate postural capacity in individuals with ASD compared with different populations matched on the degree of intellectual disability.

## 5. Conclusions

In conclusion, our findings support the applicability of the PPA in TD children and adolescents. Moreover, the PPA was successfully administered to a subgroup of age-matched individuals with ASD.

The normative data provided for the TD children and adolescents can be used in clinical and research settings for assessing postural capacity. Although preliminary, the subset data from participants with ASD may contribute to the understanding of postural impairments in developmental disorders. Interestingly, we found a reduced balance performance and age-related development progress decreased in individuals with ASD. Further studies in a larger sample size should investigate sensorimotor integration for postural control using the PPA in individuals with ASD as well as study each postural domain across age groups and ASD severity.

## Figures and Tables

**Table 1:** Demographic and clinical characteristics of ASD subjects.

ID	Age (yrs)	Sex	Intellectual Disability ^†^	ASD Severity Level ^†^	ADOS CSS
1	6	F	mild	1	4
2	6	F	mild	1	4
3	8	M	mild	1	8
4	8	M	mild	1	7
5	8	M	moderate	2	6
6	11	M	moderate	2	8
7	11	M	moderate	2	7
8	12	M	moderate	1	5
9	12	M	moderate	2	7
10	13	M	moderate	1	4
11	14	M	moderate	1	4
12	14	M	mild	1	6
13	14	M	moderate	2	6
14	14	M	moderate	2	6
15	16	M	moderate	2	7
16	16	M	severe	3	8
17	16	M	severe	2	7
18	18	M	moderate	2	6

Note: ADOS CSS = Autism Diagnostic Observation Schedule Calibrated Severity Score; M = male; F = female; ASD = autism spectrum disorder; ^†^ = intellectual disability and level of severity of autism according to DSM-5 criteria.

**Table 2:** Correlations between individual test performance and age.

	ASD	TD
	Rho Spearman	Rho Spearman
Visual acuity high-contrast	−0.254	−0.415 **
Visual acuity low-contrast	−0.021	−0.455 **
Edge contrast sensitivity	0.069	0.066
Depth perception	0.273	−0.294 **
Proprioception	−0.072	−0.053
Tactile sensitivity	0.219	0.275 **
Ankle dorsiflexion force	0.338	0.677 **
Knee extensor muscle force	0.383	0.765 **
Knee flexors muscle force	0.361	0.883 **
Reaction time: hand	0.105	−0.698 **
Reaction time: foot	−0.058	−0.770 **
Sway floor eyes open	0.320	−0.040
Sway floor eyes closed	0.330	−0.123
Sway foam eyes open	0.317	−0.319 **
Sway foam eyes closed	0.140	−0.218 *
Coordinated stability test	−0.565 *	−0.596 **
Maximum balance range test	−0.298	0.288 **

Note: ASD = autism spectrum disorder; TD = typically developing; * = correlation significant with *p* < 0.05; ** = correlation significant with *p* < 0.01.

## Data Availability

The data that support the findings of the current study are available from the corresponding author [CP] upon reasonable request.

## Supplementary Materials

The following are available online at https://www.mdpi.com/2076-3425/10/10/681/s1. Table S1: percentiles for typically developed children and adolescents.
