# Supplementary material for: Physiological Profile Assessment of Posture in Children and Adolescents with Autism Spectrum Disorder and Typically Developing Peers
Source: Brain Sci. 2020 Sep 27;10(10):681. doi: 10.3390/brainsci10100681 (PMC7601261; doi:10.3390/brainsci10100681)
Supplement: Supplementary file 1 [file brainsci-10-00681-s001.pdf]

Supplementary Table S1. Physiological Profile Assessment percentiles in typically developing children and adolescents. The five physiological subsets are presented. For all items scoring over the 90th percentile indicates poorer performance, Items scoring under the 10th percentile indicates better performance.

Vision	Percentile	Age (years)							
		6	8	11	12	13	14	16	18
**Visual acuity- high contrast**	**10**	0.93	0.92	0.70	0.87	0.80	0.83	0.68	0.81
	**50**	1.05	1.06	0.99	0.95	1.00	0.93	0.93	0.93
	**90**	1.17	1.17	1.09	1.16	1.30	1.11	1.04	1.05
**Visual acuity- low contrast**	**10**	1.22	1.12	1.18	1.14	1.08	1.08	1.03	1.07
	**50**	1.32	1.28	1.30	1.34	1.35	1.17	1.21	1.15
	**90**	1.46	1.38	1.39	1.71	3.67	1.41	1.32	1.34
**Edge contrast sensitivity***	**10**	23.80	24.00	24.00	24.00	24.00	24.00	24.00	24.00
	**50**	22.00	22.50	22.00	22.00	21.50	23.00	21.00	23.00
	**90**	19.40	19.00	19.20	19.60	20.00	21.00	18.70	21.00
**Depth perception**	**10**	0.76	0.26	0.49	0.55	0.53	0.25	0.38	0.33
	**50**	1.20	1.08	0.80	0.83	0.85	0.60	0.69	0.83
	**90**	5.00	2.70	1.79	1.34	3.85	1.63	1.87	1.38

Note: * indicates percentile scale was inverted, i.e., for all items a score over 90th percentile is an indicator of a worse performance.

Peripheral sensation	Percentile	Age (years)							
		6	8	11	12	13	14	16	18
**Proprioception**	**10**	0.84	1.14	0.64	0.00	0.60	0.30	0.76	1.00
	**50**	1.80	2.00	1.60	1.20	1.60	1.70	1.60	2.00
	**90**	4.16	3.90	3.16	3.18	8.10	3.40	3.68	3.20
**Tactile sensitivity**	**10**	3.22	3.22	3.22	3.07	3.22	3.22	3.22	3.22
	**50**	3.22	3.22	3.84	3.84	3.96	3.53	3.84	3.84
	**90**	3.84	3.84	4.08	4.08	4.43	4.08	4.56	4.08

Lower limb muscle strength	Percentile	Age (years)							
		6	8	11	12	13	14	16	18
**Ankle dorsiflexion force***	**10**	8.00	9.90	11.80	14.80	22.50	18.00	18.20	22.00
	**50**	6.00	7.00	8.00	6.50	12.00	12.00	14.00	15.00
	**90**	3.00	3.70	4.00	4.00	7.00	7.50	7.80	11.00
**Knee extension force***	**10**	22.00	25.20	35.20	43.00	56.50	47.00	87.40	78.00
	**50**	12.00	16.00	26.00	27.00	41.00	34.00	41.00	54.00
	**90**	5.20	8.10	17.60	17.20	20.50	23.00	20.00	42.00
**Knee flexion force***	**10**	10.00	15.80	20.20	21.40	29.00	33.00	43.00	52.00
	**50**	7.00	10.00	15.00	16.50	24.00	27.00	28.00	36.00
	**90**	5.20	7.40	12.20	10.30	16.50	20.00	23.60	22.00

Note: * indicates percentile scale was inverted, i.e., for all items a score over 90th percentile is an indicator of a worse performance.

Reaction time	Percentile	Age (years)							
		6	8	11	12	13	14	16	18
**Reaction time-hand**	**10**	299.00	231.00	205.60	196.10	195.50	199.50	189.90	183.00
	**50**	377.00	312.50	279.00	233.00	225.50	232.50	214.00	204.00
	**90**	472.40	348.60	328.00	328.20	282.50	265.50	236.90	236.00
**Reaction time-foot**	**10**	448.20	371.20	289.60	271.30	242.50	263.50	243.30	218.00
	**50**	527.00	450.00	336.00	314.00	288.00	307.50	279.00	270.00
	**90**	582.80	560.30	471.80	377.10	342.50	346.00	318.00	301.00

Balance	Percentile	Age (years)							
		6	8	11	12	13	14	16	18
**Sway on floor-eyes open**	**10**	205.20	43.50	80.00	52.20	48.50	188.00	58.50	126.00
	**50**	841.00	354.00	336.00	315.00	320.50	607.00	310.00	342.00
	**90**	3002.20	2926.30	1190.40	2285.40	532.00	1695.00	1419.20	816.00
**Sway on floor-eyes closed**	**10**	511.60	165.20	75.00	75.40	78.00	97.50	52.20	144.00
	**50**	1360.00	559.00	480.00	253.00	171.50	666.00	322.00	544.00
	**90**	4398.00	5613.60	1564.00	2732.10	1085.00	2329.00	2073.40	1755.00
**Sway on foam-eyes open**	**10**	480.60	540.80	175.40	151.20	69.50	484.00	127.10	252.00
	**50**	2250.00	1822.00	1155.00	757.00	1075.50	1200.00	738.50	812.00
	**90**	8857.40	11657.30	3101.20	4116.40	2717.00	3170.00	2644.20	1505.00
**Sway on foam-eyes closed**	**10**	888.00	1232.90	269.80	207.80	173.00	1189.50	315.70	580.00
	**50**	4620.00	2940.00	2394.00	2649.00	1419.00	3181.50	1816.50	1739.00
	**90**	19444.40	7469.00	8032.40	11718.80	4370.00	7524.00	3808.80	5589.00
**Coordinated stability test**	**10**	9.00	1.70	1.00	0.00	0.50	0.50	0.00	0.00
	**50**	22.00	11.00	5.00	2.00	2.50	2.00	2.00	1.00
	**90**	34.20	31.30	13.60	13.20	5.50	8.00	4.50	7.00
**Maximum balance range test***	**10**	264.78	248.50	296.00	279.28	327.71	309.18	295.22	291.40
	**50**	198.48	220.75	261.66	240.49	253.75	261.59	237.05	252.00
	**90**	121.63	178.09	170.69	202.08	198.21	191.78	199.36	188.16

Note: * indicates percentile scale was inverted, i.e., for all items a score over 90th percentile is an indicator of a worse performance.
